# Preparation of Multifunctional N-Doped Carbon Quantum Dots from *Citrus clementina* Peel: Investigating Targeted Pharmacological Activities and the Potential Application for Fe^3+^ Sensing

**DOI:** 10.3390/ph14090857

**Published:** 2021-08-27

**Authors:** Silvija Šafranko, Anamarija Stanković, Sugato Hajra, Hoe-Joon Kim, Ivica Strelec, Maja Dutour-Sikirić, Igor Weber, Maja Herak Bosnar, Petra Grbčić, Sandra Kraljević Pavelić, Aleksandar Széchenyi, Yogendra Kumar Mishra, Igor Jerković, Stela Jokić

**Affiliations:** 1 Faculty of Food Technology Osijek, University of Osijek, Franje Kuhača 18, 31000 Osijek, Croatia; silvija.safranko@ptfos.hr (S.Š.); ivica.strelec@ptfos.hr (I.S.); 2 Department of Chemistry, University of Osijek, Ulica cara Hadrijana 8/A, 31000 Osijek, Croatia; astankovic@kemija.unios.hr; 3 Department of Robotics Engineering, Daegu Gyeongbuk Institute of Science & Technology (DGIST), Daegu 42988, Korea; sugatohajra@dgist.ac.kr (S.H.); joonkim@dgist.ac.kr (H.-J.K.); 4 Division of Physical Chemistry, Ruđer Bošković Institute, 10000 Zagreb, Croatia; maja.dutour.sikiric@irb.hr; 5 Division of Molecular Biology, Ruđer Bošković Institute, 10000 Zagreb, Croatia; iweber@irb.hr; 6 Division of Molecular Medicine, Ruđer Bošković Institute, 10000 Zagreb, Croatia; mherak@irb.hr; 7 Department of Biotechnology, University of Rijeka, Radmile Matejčić 2, 51000 Rijeka, Croatia; petra.grbcic@biotech.uniri.hr; 8 Faculty of Health Studies, University of Rijeka, Viktora Cara Emina 5, 51000 Rijeka, Croatia; sandrakp@uniri.hr; 9 Institute of Pharmaceutical Technology and Biopharmacy, Faculty of Pharmacy, University of Pécs, H-7624 Pécs, Hungary; szealex@gamma.ttk.pte.hu; 10 Mads Clausen Institute, NanoSYD, University of Southern Denmark, Alsion 2, 6400 Sønderborg, Denmark; mishra@mci.sdu.dk; 11 Department of Organic Chemistry, Faculty of Chemistry and Technology, University of Split, Ruđera Boškovića 35, 21000 Split, Croatia

**Keywords:** citrus waste, carbon quantum dots, N-doping, Fe^3+^ detection, biocompatibility

## Abstract

Carbon quantum dots (CQDs) have recently emerged as innovative theranostic nanomaterials, enabling fast and effective diagnosis and treatment. In this study, a facile hydrothermal approach for N-doped biomass-derived CQDs preparation from *Citrus clementina* peel and amino acids glycine (Gly) and arginine (Arg) has been presented. The gradual increase in the N-dopant (amino acids) nitrogen content increased the quantum yield of synthesized CQDs. The prepared CQDs exhibited good biocompatibility, stability in aqueous, and high ionic strength media, similar optical properties, while differences were observed regarding the structural and chemical diversity, and biological and antioxidant activity. The antiproliferative effect of CQD@Gly against pancreatic cancer cell lines (CFPAC-1) was observed. At the same time, CQD@Arg has demonstrated the highest quantum yield and antioxidant activity by DPPH scavenging radical method of 81.39 ± 0.39% and has been further used for the ion sensing and cellular imaging of cancer cells. The obtained results have demonstrated selective response toward Fe^3+^ detection, with linear response ranging from 7.0 µmol dm^−3^ to 50.0 µmol dm^−3^ with *R*^2^ = 0.9931 and limit of detection (LOD) of 4.57 ± 0.27 µmol dm^−3^. This research could be a good example of sustainable biomass waste utilization with potential for biomedical analysis and ion sensing applications.

## 1. Introduction

Over the last decade, there has been growing interest in developing biocompatible fluorescent nanomaterials which could represent a safe substitute to the toxic metal luminescent nanoparticles. Due to their excellent optical properties, carbon dots have gained considerable attention with benefits to biomedical and biological applications. Carbon quantum dots (CQDs) have been extensively studied for their biocompatibility, wavelength-tunable emission, relatively facile synthesis, and photostability, while doping and surface functionalization endow the CQDs with remarkable potential for a broad spectrum of applications [1]. The CQDs have been considered as “zero-dimensional“ nanoparticles, commonly prepared by a “top-down“ or “bottom-up“ approach [2]. The “bottom-up“ approach has gained considerable attention for the facile and efficient fabrication of nanoparticles by the carbonization process of organic precursors through solvothermal, hydrothermal, microwave, and ultrasonication treatments [3]. Among the “bottom-up“ approaches, hydrothermal synthesis is the most commonly used method for obtaining CQDs due to its simplicity and outstanding efficiency in obtaining water-soluble materials with high quantum yield (QY) [4]. 

The waste produced during processing in the food industry has become a burgeoning problem, making the proper waste management and its utilization of great importance for the environment and public health [5]. Citrus fruits are highly produced and consumed worldwide, and it has been reported that 110–120 million tons of citrus waste is generated every year by the food processing industry [6]. The most recent studies have shown that waste biomass disposal can develop high-value products used in the food, pharmaceutical, and cosmetic industry, and for medical purposes. The extraction and purification of bioactive and health-beneficial compounds derived from the waste biomass have been extensively studied. It is well known that natural biomass can be converted into valuable products usable in a wide variety of applications [7]. Plant-based biomass mainly comprises of carbohydrates, proteins, lipids, minerals, and phenolic compounds, and therefore it could represent an excellent carbon source for CQDs fabrication.

Moreover, several studies have used biomass waste for obtaining CQDs, including citrus [8,9], kitchen waste [10], mango peel [11], banana peel [12], expired milk [13], egg shell [14], and agarose waste [15], all exhibiting good chemical and optical properties, and obtained carbon dots were applicable for the bioimaging, biosensing, and environmental monitoring. A significant challenge in the fabrication of biomass-derived CQDs represents obtaining CQDs with a high quantum yield. The enhancement of photoluminescent properties could be achieved by surface modification, which was previously demonstrated by the strong correlation between photoluminescence and surface defects of the material [16,17]. Heteroatom-doping of pristine CQDs could also improve photoluminescence properties by increasing surface-state defects and chemical reactivity, creating reactive groups on the surface [18]. Nitrogen (N)-doping is the most commonly applied for improving the performance of CQDs. Previously, Qi et al. [19] have reported the facile preparation of N-doped CQDs using rice residue as a carbon precursor and amino acid lysine as nitrogen dopant. The synthesized N-CQDs were used as a fluorescent probe for Fe^3+^ and tetracycline detection with good selectivity and sensitivity. Moreover, Gao et al. [8] synthesized N-doped CQDs via the hydrothermal procedure in ammonia using orange and watermelon peel as carbon precursors, which were finally used for the oxytetracycline detection and analysis in water monitoring. 

Detection of ferric (Fe^3+^) state of iron is of great importance for biological systems as it has a crucial role in biochemical pathways of the living cells [20]. It is well known that imbalance in iron homeostasis may cause many health disorders and medical conditions such as anemia, neurodegenerative disease (e.g., Parkinsonism, Alzheimer’s disease), hemochromatosis, etc. [21]. The standard methods for iron detection in biological samples (e.g., blood, urine) typically include inductively coupled plasma mass spectrometry (ICP-MS) and colorimetric assays, as well as ion chromatography [22]. Therefore, novel and inexpensive methods for Fe^3+^ detection are undoubtedly a topic of interest. 

This study aimed to prepare N-doped CQDs via a facile hydrothermal approach using clementine (*Citrus clementina*) peel as the carbon source and amino acids glycine (Gly) and arginine (Arg) as nitrogen dopants. In order to compare the properties and bioactivity of synthesized CQDs, three different samples have been prepared: pure CQDs without N-doping (pristine CQDs), CQD@Gly using N-dopant Gly, and CQD@Arg using Arg as N-dopant with a higher content of nitrogen compared to Gly. A detailed chemical, structural, and optical characterization of prepared CQDs has been further investigated, showing good biocompatibility as well as antioxidant and biological activity, depending on the degree of surface functionalization. The prepared CQDs showed good selectivity, sensitivity, and potential application toward fluorescent Fe^3+^ detection, also showing great potential of the prepared CQDs in cellular imaging.

## 2. Results and Discussion

The preparation of CQDs from *Citrus clementina* peel is an example of the efficient utilization of biomass waste for the development of nanomaterials with potential applications in biomedical analysis and ion sensing. It is well known that citrus peel is rich in essential oils, carbohydrates (soluble sugars), phenolic compounds, and minerals [23]. Carbon quantum dots derived from organic precursors undergo five main phases: thermal decomposition, condensation, polymerization, carbonization, and surface passivation [24,25]. By adding amino acids Gly and Arg into the mixture, a condensation reaction of Maillard non-enzymatic reaction could take place. The reactive carbonyl groups of the carbohydrates react with the nucleophilic amino groups of amino acids, and through the array of the chemical reactions, characteristic Maillard reaction products are formed. In order to compare different properties of synthesized CQDs, three different materials were prepared: pure CQD using only citrus peel as carbon precursor, CQD@Gly using a citrus peel and amino acid Gly, and CQD@Arg using citrus peel as carbon source and amino acid Arg as N-dopant. Firstly, the differences were observed in the colour of samples, as samples with added amino acid had a characteristic brown colour (Table S1) and produced a burnt, caramel-like odour. 

The most common method for CQDs purification is dialysis using membrane tubings [18,26,27]. However, the efficiency of the dialysis procedure is more or less disputable. Chen et al. [28] reported the HPLC protocol to evaluate the efficiency of the CQDs purification using dialysis and concluded that most of the CQDs in published works were inadequately dialyzed. Hence, we have assessed the efficiency of the applied protocol using dialysis membrane MWCO 1kDa by recording HPLC chromatograms of the retentate, and results are presented in Figure S1. As it can be seen, the as-prepared pure CQDs show multiple peaks as a result of the CQDs contamination found at the ~30–50 min, mainly contributed by the unreacted chemical species formed during thermal treatment. The retention times of the low absorption peaks are 5.89–17.20 min, which could appear due to the presence of the CQDs by-products of different structural properties. After 24 h, a significant decrease in the number of absorption peaks corresponding to the by-products of CQDs has been observed at the retention time of ~30–50 min. Finally, 48 h was found to be adequate dialysis time for removing the majority of by-products, as significant improvement in reducing the number of absorption peaks was observed during analysis. 

**Figure 1:**
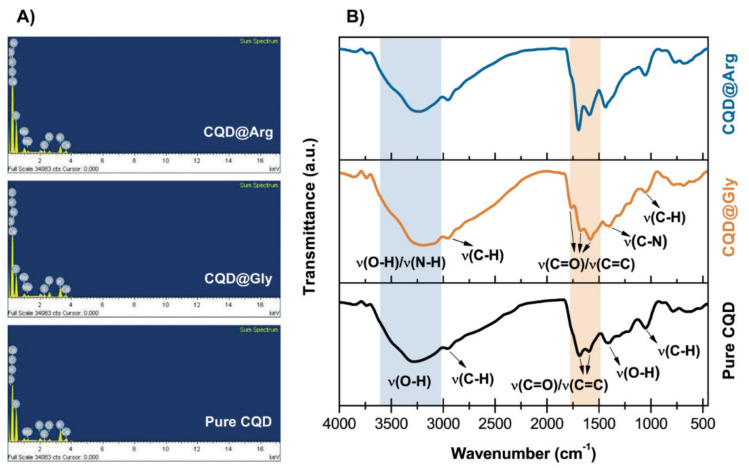
EDS spectrum (**A**) and FTIR spectra (**B**) of prepared CQDs and N-CQDs.

From a chemical aspect, pure CQD were prepared as a reference system consisting mainly of carbon (61.89%) and oxygen (34.74%), as demonstrated by EDS analysis (Figure 1A, Table S2), while nitrogen was not detected in the sample. The addition of amino acids Gly and Arg resulted in nitrogen incorporation in the prepared CQDs, as shown in Table S2, Figure 1A, and Figure S2.

Furthermore, the EDS spectrum and summarized elemental composition of the prepared CQDs demonstrated the presence of carbon and oxygen in all samples. However, elements such as calcium (Ca), magnesium (Mg), and potassium (K) could also be found in traces as citrus peels contain significant amounts of minerals. Introduction of amino acids to the reaction mixture resulted in nitrogen content increase from 3.07% in atomic ratio for CQD@Gly, and 4.87% in atomic ratio for CQD@Arg (Table S2 and Figure S2). In order to better understand the processes occurring on the surface of CQD, zeta (ζ)-potential was measured for all prepared samples. The results indicated that *ζ*-potential of pure CQD was −31.3 ± 2.4 mV, while −21.0 ± 3.2 mV and -12.7 ± 2.1 mV for CQD@Gly and CQD@Arg, respectively. The changes in the ζ-potential of N-doped CQDs to the less negative values could result from the surface passivation by adding amino acids to the mixture. It could be assumed that pure CQDs contain –COOH functional groups on the surface, making ζ-potential more negative. By adding amino groups to the surface of CQDs, the ζ-potential shifted to less negative values. 

The presence of the main functional groups was demonstrated by FTIR spectroscopy measurements, as shown in Figure 1B. The common characteristics for all prepared CQDs are the broad peaks at approximately 3267–3305 cm^−1^ which indicated the existence of O-H and N-H groups and the presence of functionalized groups [26,27]. The strong peaks centred at 1680 cm^−1^ and 1041 cm^−1^ could be attributed to C=O and C-O vibration modes. The existence of the strong peak centred at 1580 cm^−1^ could indicate the presence of unsaturated C=C vibrations formed within the carbon core of the CQDs, and the peaks at 1417 cm^−1^, 1400 cm^−1^, and 1380 cm^−1^ could be assigned to O-H, C-N, and C-H bending vibration modes, respectively [11,29,30]. The weak peaks between 674 cm^−1^ and 640 cm^−1^ could indicate the presence of C-H bonds. 

**Figure 2:**
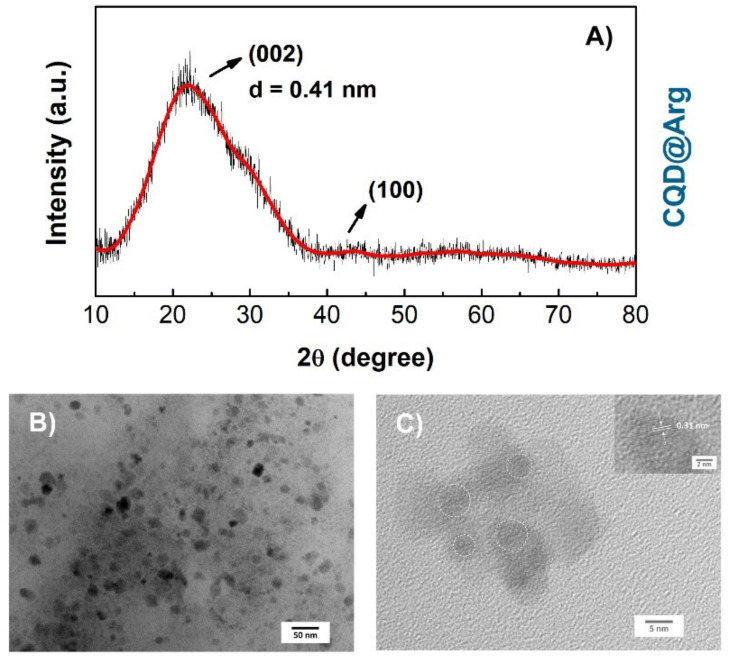
(**A**) XRD pattern of CQD@Arg, (**B**) TEM images showing CQD@Arg nanoparticles, and (**C**) HR-TEM image showing lattice spacing of *d* = 0.31 nm.

The XRD measurements of the prepared CQDs did not show significant differences between patterns (Figure 2A and Figure S3). The broad diffraction peak at 2*θ* = (22.28 ± 0.43)° corresponds to the (002) plane, demonstrating the amorphous nature of synthesized CQDs. The broader interlayer spacing of (0.40 ± 0.01) nm compared to 0.34 nm of graphite also indicates the amorphous nature and low crystallinity of CQDs caused by the surface functionalization [31]. The low-density peak at 2*θ* = (43.30 ± 0.22)° corresponds to the (100) plane, also indicating the presence of graphitic carbon [8,32,33].

The average particle size and morphological characteristics of the CQD@Arg sample were determined by the transmission electron microscopy (TEM) and high-resolution TEM (HR-TEM) analysis. As it can be seen from Figure 2B, TEM images show nearly spherical morphology of synthesized CQD@Arg particles with an average diameter of (8.52 ± 1.72) nm and the existence of aggregates giving particles of diameter ≥10 nm. The HR-TEM (Figure 2C) images show lattice spacing of *d* = 0.31 nm.

### 2.1. Optical Characterization

**Figure 3:**
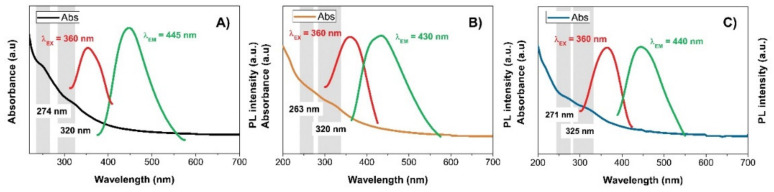
UV-vis absorption spectrum with maximum fluorescence excitation and emission spectrum of (**A**) Pure CQD, (**B**) CQD@Gly, and (**C**) CQD@Arg.

Figure 3A–C shows the UV-visible absorption spectra of the prepared CQDs. As it can be seen, in the UV region, there are two weak absorption peaks present at around 270 nm designated for π–π^*^ transition of C=C double bond, and around 320 nm for n–π^*^ transition of C=O bonds. These results are in agreement with the above-discussed data and analysis. 

**Figure 4:**
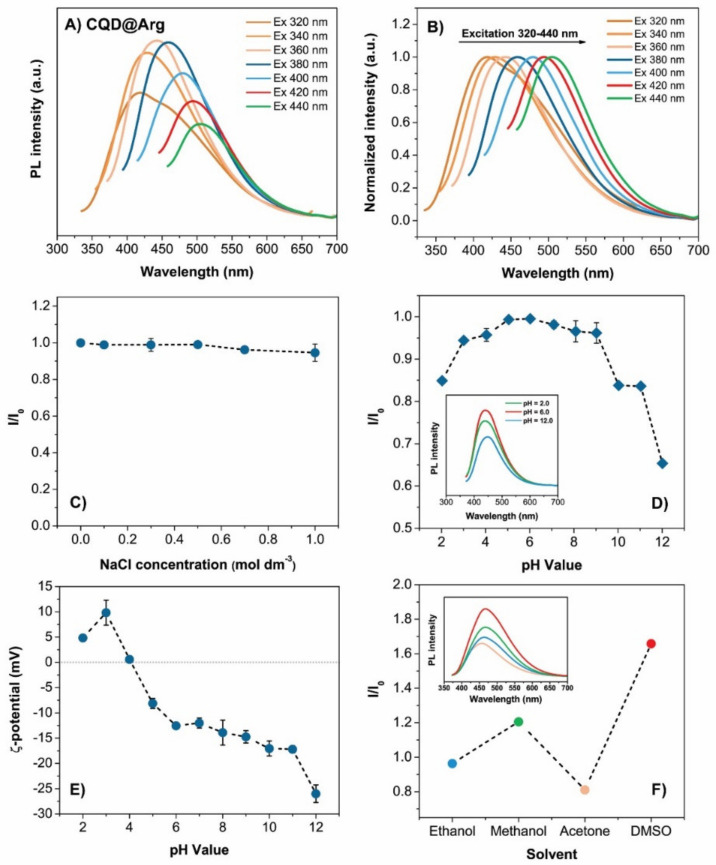
Optical characterization of CQD@Arg: (**A**) Fluorescence emission spectra of CQD@Arg at different excitation wavelengths ranging from 320 nm to 440 nm (increments of 20 nm); (**B**) normalized fluorescence emission intensity, (**C**) stability of CQD@Arg in the high ionic medium of NaCl (0–1.0 mol dm^−3^; λ_Ex_ = 360 nm); (**D**) fluorescence intensity vs. pH variation (λ_Ex_ = 360 nm); (**E**) variations of *ζ*-potential as a function of pH; (**F**) fluorescence spectra of CQD@Arg in ethanol, methanol, acetone, and DMSO (λ_Ex_ = 360 nm).

The fluorescence measurements showed the maximum emission for the pure CQD at λ_EM_ = 445 nm and excitation wavelength of λ_EX_ = 360 nm, at λ_EM_ = 430 nm for CQD@Gly, and at λ_EM_ = 440 nm for CQD@Arg, at the same excitation wavelength of λ_EX_ = 360 nm. The calculated quantum yields of all prepared samples were as follows: 1.17 ± 0.12% for pure CQD, 1.53 ± 0.23%, and 2.17 ± 0.11% for CQD@Gly and CQD@Arg, as shown in Figure S4, demonstrating also a statistically significant differences (*p* < 0.05) among the prepared samples of carbon dots. This indicates that an increase in the quantum yield results from the nitrogen passivation on the carbon quantum dots surface. In general, the literature reports show that biomass-derived CQDs have lower quantum yield and that surface passivation plays an essential role in improving their optical and chemical properties [34]. Therefore, the material with the best-determined quantum yield, precisely CQD@Arg, was used for further investigation and analysis. Furthermore, fluorescence spectra were recorded in order to depict an excitation-dependent behaviour of all prepared samples. As it can be observed (Figure S5), with an increase in excitation wavelength from 320 nm to 440 nm, the emission maximum for pure CQD shifts from 428 nm to 495 nm. In the case of the CQD@Gly, the shift of the emission maximum is recorded from 400 nm to 493 nm (Figure S6), and, for CQD@Arg, from 418 nm to 508 nm (Figure 4A). However, this observation was followed by a concurrent decrease in the emission intensity of analysed samples. The stability of CQD@Arg in NaCl systems of different concentrations (*c*(NaCl) = 0–1.0 mol dm^−3^) was also investigated, and the results indicated that samples show significant stability in high ionic media, as can be noted by the minimal changes in the fluorescence intensity with the increase in salt concentration (Figure 4C). The same observation was denoted for the pure CQD and CQD@Gly, demonstrating their good stability in high salt conditions, the results of which are shown in Figures S5c and S6c. However, the PL behaviour was strongly dependent on the pH of the medium containing CQDs in the pH range from pH = 2.0 to pH = 12.0, and the surface charge of CQDs versus adjusted pH media was explained by performing ζ-potential measurements for CQD@Arg sample. As it can be seen in Figure 4D,E, there were no significant changes in the fluorescence intensity of the CQD@Arg sample over the pH range from pH = 6.0 to pH = 10.0, while a significant decrease in fluorescence intensity was recorded at extreme values of pH, more precisely at pH = 2.0 and pH=12.0. Some changes occurred in acidic conditions from pH = 2.0 to pH = 4.0, and also a decrease in fluorescence intensity was observed at pH = 10.0. The measurements of *ζ*-potential as a function of pH showed changes in ζ-potential from 9.8 ± 2.5 mV to −26.0 ± 1.7 mV, indicating that changes occurred on the surface states of prepared CQDs, most likely due to the ionization of the surface functional groups. From the *ζ*-potential vs. pH diagram, the isoelectric point of CQD@Arg (pH_iep_) was assessed to be pH_iep_ = 4.10 (Figure 4E). At the acidic conditions (lower pH) below the isoelectric point, CQDs are protonated and have a positive net charge on the surface, while, with an increase in the pH, above pH_iep_ = 4.10, in the alkaline conditions, CQDs form a negative net charge on the surface due to the deprotonation of carboxylic groups. Similar pH behaviour is demonstrated and presented in Figures S5d and S6d for pure CQD and CQD@Gly, respectively. Finally, the effect of different solvents on the fluorescence intensity of CQD@Arg was investigated (Figure 4F). The CQD@Arg showed good dispersibility in the polar protic solvents, such as ethanol and methanol; however, lower dispersibility was observed in the polar aprotic solvent acetone. The interesting observation was made by good dispersibility of CQD@Arg nanoparticles in DMSO solvent exhibiting the highest emission intensity with more significant red shift (~27 nm) among the investigated solvents. The fluorescence intensities of CQDs dispersed in alcohols were lower and, in DMSO, were slightly higher compared to intensities recorded in water. The acidic nature of the prepared CQDs could contribute to this solvent-dependent behaviour, as it was previously demonstrated that hydrogen donating-accepting effects between CQDs and solvents could play a key role [35]. The images of all prepared blue-emitting CQDs under daylight and UV light irradiation (365 nm) are provided in Table S1, showing potential application of prepared CQDs as fluorescent ink as well.

### 2.2. Cell Viability and Cytotoxicity

The antiproliferative effects of pure CQD, CQD@Arg, and CQD@Gly are presented in Table 1. The LC_50_ values were above the tested concentrations. Antiproliferative effects were not observed for samples of pure CQD and CQD@Arg on tested tumour cell lines (Table 1). However, CQD@Gly selectively inhibited the growth of the CFPAC-1 carcinoma cell line (IC_50_ = 6.91 ± 0.81 µg/mL) as it did not affect the growth of HepG2, MCF-7, and HCT-116 tumor cell lines at tested concentrations. This selectivity may be attributed to the ability of CQD@Gly to enter CFPAC-1 cells at a higher rate or to the specific genetic properties of cells. All tested samples uniformly inhibited the growth of the non-transformed human fibroblasts HFF-1 cells. However, contrary to the observed effects on tumour cell lines that were highly similar and reproducible between biological experiments, CQD@Arg and CQD@Gly samples induced the proliferation of HFF-1 in one of three biological experiments. The results are accordingly presented separately (Table 1). The observed variability in the proliferation of HFF-1 cells might be a consequence of the antioxidative effects of CQD@Arg and CQD@Gly. Indeed, small changes in ROS levels are essential in cell proliferation, and ROS levels depend on the cell cycle phase [36]. The effect on the redox status of a normal cell may, thus, have different outcomes depending on the cell cycle phase in which the treatment was applied. 

### 2.3. Determination of Antioxidant Activity

**Figure 5:**
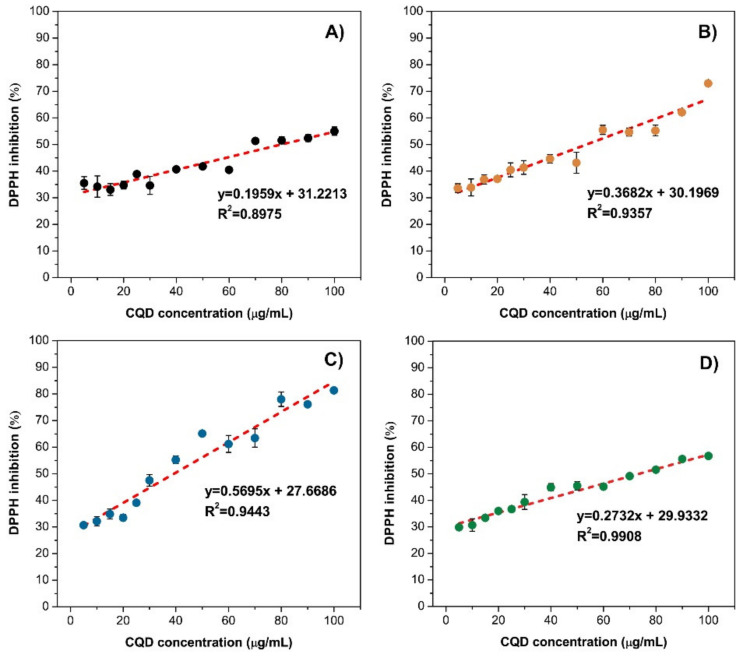
Antioxidant activity of prepared CQDs using DPPH free radical assay for (**A**) pure CQD; (**B**) CQD@Gly; (**C**) CQD@Arg, and (**D**) *Citrus clementina* extract.

The DPPH assay has been employed in order to determine the antioxidant activity of the synthesized CQDs samples. Commonly, antioxidant activity is a parameter used to evaluate the ability of the material and/or prepared sample to scavenge and neutralize free radicals. The antioxidant activity could be determined by measuring the changes in colour from purple to yellow, as the antioxidant interacts with the DPPH free radical. For this work, different concentrations of synthesized CQDs were prepared in the concentration range from 0 µg/mL to 100 µg/mL, then were added to the 50-µM methanolic solution of DPPH, and absorbance was measured at 517 nm. As shown in Figure 5, the highest antioxidant activity was determined in the CQD@Arg with the scavenging activity of 81.39 ± 0.39% at the concentration of 100 µg/mL, while the lowest was determined in the pure CQDs of 55.04 ± 1.59% at the concentration of 100 µg/mL. The concentration of the sample that caused a 50% decrease in the DPPH activity is defined as half-maximal effective concentration (EC_50_), and, in this case, was determined according to the equation obtained by linear regression. For the pure CQD, the EC_50_ was calculated to be 79.83 ± 1.72 µg/mL (*R*^2^ = 0.8975), while, for CQD@Gly and CQD@Arg, they were determined to be EC_50_ = 53.78 ± 0.97 µg/mL (*R*^2^ = 0.9357) and EC_50_ = 39.21±0.41 µg/mL (*R*^2^ = 0.9443), respectively.

The antioxidant activity of pure CQD is comparable to the activity of aqueous *Citrus clementina* extract obtained by ultrasound-assisted extraction technique (Figure 5D). It is well-known that extracts of citrus peel exhibit diverse biological activities due to the presence of different phenolic compounds, and therefore it could be used for the purpose of antioxidant activity assessment [7]. The obtained EC_50_ of *Citrus clementina* extract was calculated to be 73.45 ± 1.12 µg/mL (*R*^2^ = 0.9908), while as previously denoted, the EC_50_ for pure CQD was determined to be 79.83 ± 1.72 µg/mL. However, the EC_50_ values for both CQD@Gly and CQD@Arg are determined to be lower demonstrating higher antioxidant activity compared to the activity of *Citrus clementina* extract and pure CQD. This indicates that the antioxidant activity could be enhanced by both –COOH and –NH_2_ functional groups on the surface, showing the strong structure–activity relationship [37].

### 2.4. CQD@Arg for Ion Sensing and Fe^3+^ Detection

**Figure 6:**
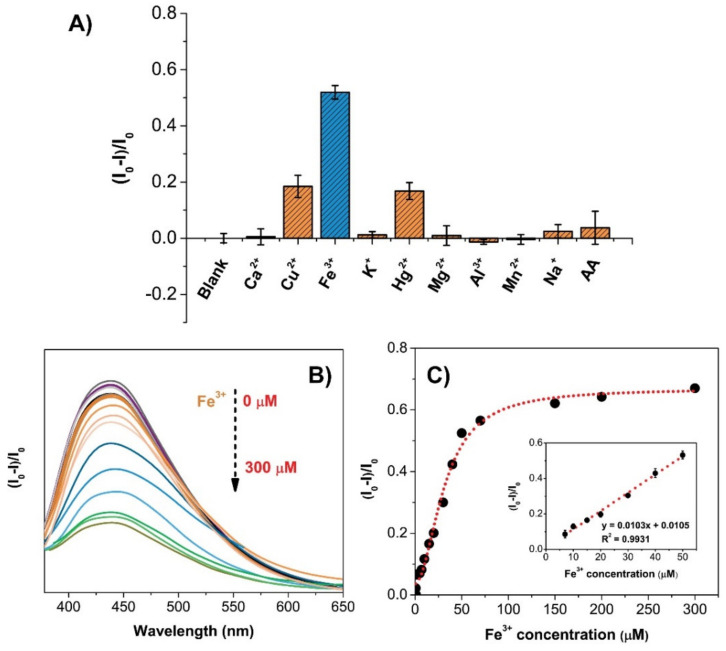
(**A**) Fluorescence response of CQD@Arg to different metal ions and ascorbic acid, (**B**) the fluorescence spectral quenching upon the addition of different Fe^3+^ concentrations (0.5–300 μmol dm^−3^); and (**C**) the relative fluorescence response (*I*_0_-*I*)/*I*_0_ of CQD@Arg with the Fe^3+^ addition confirming exponential behaviour (inset: linear response ranging from 7.0 to 50.0 μmol dm^−3^).

In order to evaluate selectivity and interference effects in ion sensing and detection using CQD@Arg, different ions were tested, including Ca^2+^, Cu^2+^, Fe^3+^, K^+^, Hg^2+^, Mg^2+^, Al^3+^, Mn^2+^, Na^+^, as well as sensitivity on the ascorbic acid (AA). For this purpose, the concentration of CQD@Arg was adjusted to 25 µg/mL, and the concentration of different chemical species was prepared to 100 µM. The results indicated high sensitivity and selectivity toward Fe^3+^ ions detection, and the coexistence of other tested ions resulted in a negligible interfering effect on the fluorescence quenching (Figure 6A). The noticeable fluorescence quenching was measured with the addition of Fe^3+^ ions (the fluorescence intensity was reduced to >50% compared to blank), and slight fluorescence quenching was observed with the addition of Cu^2+^ and Hg^2+^ ions (fluorescence intensity was reduced <20% compared to blank), indicating that Fe^3+^ ions had the strongest ability to quench fluorescence intensity of CQD@Arg.

Therefore, the application of CQD@Arg in the detection of Fe^3+^ ions was investigated. Different concentrations of Fe^3+^ were prepared in the concentration range from 0.5 µmol dm^−3^ to 300 µmol dm^−3^, and the fluorescence spectra were presented in Figure 6B. The concentration of as-prepared CQD@Arg was maintained at 25 µg/mL. The fluorescence intensity on the y-axis was expressed as (*I*_0_/*I*)*I*_0_ ratio, where *I*_0_ represents the fluorescence intensity of blank without the addition of Fe^3+^ ions, and *I* represents the ratio of a respective sample with different Fe^3+^ concentrations. The relationship of the recorded fluorescence intensities and Fe^3+^ concentrations could be described by an exponential function (y = 0.6992 − 0.6331/(1 + (x/31.5993)^1.94^) with an obtained good coefficient of determination of *R*^2^ = 0.9891 (Figure 6C). A linear range was determined at a lower Fe^3+^ ion concentration ranging from 7 µmol dm^−3^ to 50 µmol dm^−3^, with the obtained coefficient of determination *R*^2^ = 0.9931, the limit of detection of LOD = 4.57 ± 0.27 µmol dm^−3^, and the limit of quantification of LOQ = 15.24 ± 0.89 µmol dm^−3^ (Figure 6C).

These observations clearly indicate the strong affinity of CQDs toward Fe^3+^ ions due to the chemical nature of CQDs. Zhu et al. [38] have reported that Fe^3+^ ions most likely coordinate with amino- and oxygen-rich groups on the CQD surface and cause quenching. A more detailed mechanism is provided by Issa et al. [39], reporting that newly formed bonds between Fe^3+^ ions and oxygen/nitrogen atoms of functional groups on the CQD surface could result in migration of the electrons in the excited state to the half-filled d-orbitals of Fe^3+^ with electron configuration of [Ar]3d^5^ causing significant fluorescence quenching [39,40]. This work and developed model show promising potential for the application in analytical chemistry and biomedical analysis, including Fe^3+^ detection in biofluids [41,42]. The Fe^3+^ ion sensitivity and properties of different biomass-derived CQDs were compared with several reported works presented in Table 2.

### 2.5. Application in Bioimaging

**Figure 7:**
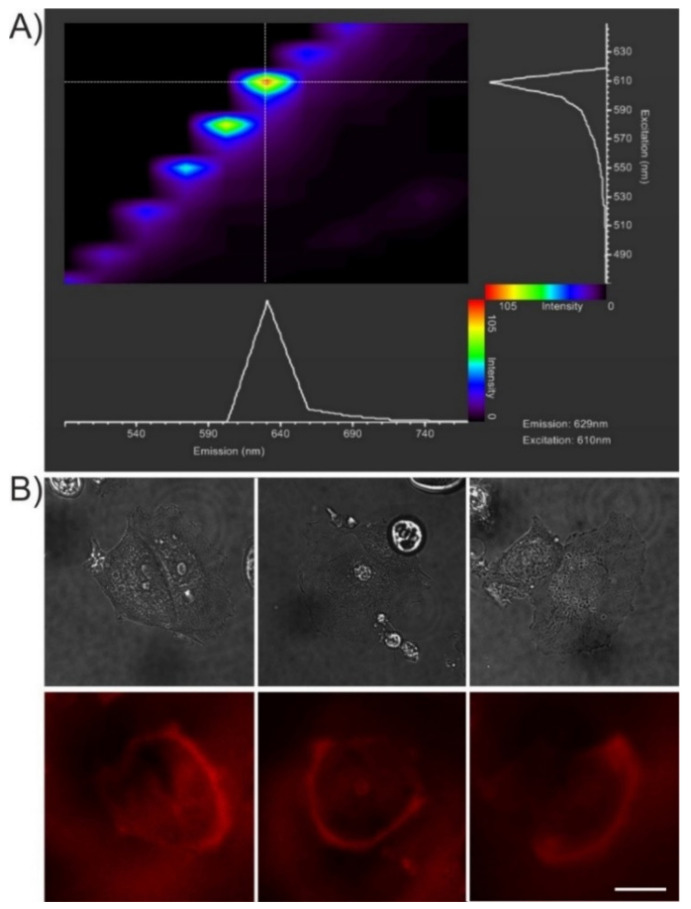
(**A**) Microspectrofluorimetry of CQD@Arg in the visible light range. The double-lambda plot of CQD@Arg adhered to the glass surface was obtained using excitation between 470–650 nm and detecting emission of fluorescence between 490–770 nm. The maximum emission was detected using excitation at 610 nm, and those conditions were used for cell imaging. (**B**) Living MCF-7 cells labelled with CQD@Arg imaged by confocal microscopy in transmission (upper row) and fluorescence (lower row) channels (λ_exc_ = 610 nm; λ_em_ = 620–690 nm). Average fluorescence intensity projections of 3D stacks covering the cell thickness are shown in the fluorescence channel: scale bar, 20 μm.

In order to test the applicability of nitrogen-doped CQDs in cell labelling and imaging, MCF-7 cells were incubated for 4 h with 10 µg/mL of CQD@Arg and imaged by confocal microscopy. To determine the optimal conditions for imaging, microspectrofluorimetry of a thin layer of CQD@Arg particles formed on the surface of the glass coverslip was performed by stepwise scanning of the excitation and detection wavelengths in the visible range from blue to far-red (Figure 7A). Based on the results of the microspectrofluorimetry, we used the 610-nm laser light for excitation and detected the fluorescence in the 620–690 nm range. The average projections of 3D stacks of three representative cells are shown in Figure 7B. CQD@Arg appears to be labelling primarily the cell plasma membrane, which is particularly apparent in the lamellar region of cells (Figure 7B). Detention of the fluorescent material at the cell surface may result from its stickiness, which also caused its adhesion to the glass surface. Indeed, the process of N-doping resulted in a significant shift in the ζ-potential of N-doped CQD@Arg particles to the less negative values compared to pure CQDs (see above).

The membrane-labelling property of the CQD@Arg particles and their fluorescence emission in the far-red region of the visible spectrum makes them an attractive candidate for applications in bioimaging. However, their adhesion to glass produces a strong fluorescent background that precludes visibility of the cellular structures at the ventral cell surface. It has been proposed that the red fluorescence of arginine-derived fluorescent CQDs can arise as a consequence of the re-absorption of primary blue fluorescence under high-density conditions, a phenomenon known as the inner filter effect [54,55]. Alternatively, the red-shifted excitation and emission spectra of CQD@Arg might result from delocalization of the excited states and collective excitations, i.e., excitons, which appear in the condensed state of the nanosized particles in the surface layers [56].

## 3. Materials and Methods

### 3.1. Chemicals and Materials

The *Citrus clementina* peels were collected from the Dalibor Ujević family farm (Opuzen, Croatia) and were washed with water several times before use. Amino acids L-glycine and L-arginine were purchased from Merck, Germany, and Sigma–Aldrich, USA. Ascorbic acid (Gram Mol, Croatia) and different inorganic salts used for selectivity measurements were as follows: FeCl_3_ × 6H_2_O (VWR International, USA), MgCl_3_ × 6H_2_O (T.T.T., Croatia), CaCl_2_ × 2H_2_O (BDH Prolabo, UK), KCl, NaCl (Gram Mol, Croatia), CuCl_2_ x 2H_2_O (Kemika, Croatia), AlCl_3_ x 6H_2_O (Brend Kraft, Germany), HgCl_2_ (VWR International, USA), and MnCl_2_ x 4H_2_O (Sigma–Aldrich, St. Louis, MO, USA). The quinine sulfate and DPPH assay (2,2′-diphenyl-1-picrylhydrazyl) were supplied by Merck, Germany. The chemicals and solvents, such as ethanol, methanol, dimethyl sulfoxide (DMSO), acetone, sulfuric acid, acetic acid, and hydrochloric acid, were purchased by Merck, Germany, while NaOH pellets were obtained by Gram Mol, Croatia. Milli-Q pure water obtained by the Milli-Q Millipore system (conductivity ≤ 0.054 μS/cm) was used throughout the samples preparation and analytical measurements. The sample purification was carried out using dialysis membrane Spectra/Por 7 (MWCO 1kDa) supplied by Spectrum Labs (Rancho Dominguez, CA, USA). 

All reagents and chemicals were of analytical grade and were used further without purification.

### 3.2. Instruments and Characterization Techniques

The structural differences between synthesized CQDs samples were investigated and recorded by Shimadzu FT-IR 8400S spectrometer (Shimadzu, Kyoto, Japan) using the DRS 8000 attachment over the 4000–400 cm^−1^ spectral region at the resolution of 4 cm^−1^. Briefly, a small amount (~1-2 mg) of the dried sample was mixed with KBr powder (IR grade), while data analysis was performed using the software package IR Solution 1.30, supplied by the manufacturer. Ultraviolet-visible (UV-Vis) absorption spectroscopy measurements were carried out using uniSPEC 2 spectrophotometer (LLG Labware, Czech Republic). X-ray powder diffraction data were obtained by means of Empyrean (Malvern PANalytical, Almelo, Netherlands) using CuKα radiation (λ = 0.1541 nm) with a step size of 2 °/min. The scanning electron microscope (Hitachi S-4800, Japan) was utilized to record the surface micrographs. The average size and morphology of nanoparticles were determined by high-resolution transmission electron microscopy (HR-TEM) using an FEI Tecnai G2 20 X Twin instrument (FEI, Hillsboro, OR, USA) operated at a 200-kV accelerating voltage. The sample was prepared by drop-casting the CQD@Arg sample onto a 200-mesh copper grid, with carbon film (EMR Carbon support grids, Micro to Nano Ltd., Haarlem, The Netherlands) and drying in a vacuum desiccator at room temperature. The chemical composition and nitrogen content were detected by the elemental dispersive X-Ray spectroscopy (Oxford Instruments, Abingdon, UK) attached to SEM. The optical and fluorescence behaviour of the prepared samples were analysed using Cary Eclipse Fluorescence Spectrophotometer (Varian Inc. Santa Clara, CA, USA). Zeta potential (ζ-) of CQDs in aqueous suspensions was determined by electrophoretic light scattering using Zetasizer Nano ZS (Malvern Instruments, Malvern, UK). The zeta potential of the particles was calculated from the measured electrophoretic mobility by means of the Henry equation using the Smoluchowski approximation (*f*(κa) = 1.5). The results are reported as an average value of the three measurements. The data processing was performed by the Zetasizer software 7.13 (Malvern Instruments, Malvern, UK). All measurements were performed at 25 °C. Laser scanning confocal microscopy was performed using a Leica TCS SP8 X laser scanning microscope equipped with an HC PL APO CS2 63×/1.40 oil objective and a supercontinuum excitation laser (Leica Microsystems, Wetzlar, Germany). Microspectrofluorimetry of CQD@Arg in the visible range was performed by recording the fluorescence intensity of a thin layer of CQD@Arg adhered to the surface of a 24 × 24 mm glass coverslip after incubating 1 mL of its 100 µg/mL solution in bidistilled water for 30 min. For microspectrofluorimetry, we used the *xzλΛ* imaging mode in the configuration with constant excitation laser power with excitation wavelengths between 470–650 nm in 10-nm steps, and detected the emission of fluorescence between 490–770 nm in 28-nm steps (detection bandwidth was 20 nm). For bioimaging, 1.5 × 10^5^ MCF-7 cells were seeded onto 35-mm 4-Chamber Glass Bottom Dish (Cellvis) in DMEM (Dulbecco’s modified Eagle’s medium, Invitrogene) supplemented with 10% fetal bovine serum (FBS, Invitrogene), 2 mM of glutamine, sodium pyruvate, 100 U/mL of penicillin, and 100 μg/mL of streptomycin. The cells were kept in a humidified chamber with 5% CO_2_ at 37 °C. After 24 h, the medium was replaced with a fresh one containing 10 µg/mL of CQD@Arg in which the cells were incubated for four hours and then rinsed three times with PBS. During bioimaging, the cells were kept in Lebovitz’s L-15 medium to support cell growth in environments without CO_2_ equilibration (Thermo Fisher Scientific, Waltham, MA, USA). The excitation wavelength and detection range used for imaging of living MCF-7 cells were 610 nm and 620–690 nm, respectively. The cells were imaged in the *xyz* mode, and optical 3D stacks were collected from 1 µm above the glass surface up to the upper cell surface. The fluorescence images are displayed as average fluorescence intensity projections of the obtained stacks.

### 3.3. Preparation of Undoped and N-doped CQDs

**Figure 8:**
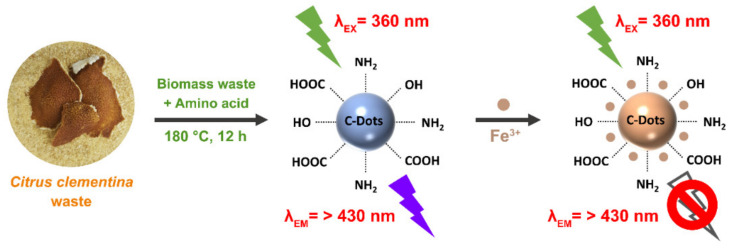
Schematic illustration of CQDs preparation and their application in Fe^3+^ sensing.

The collected *Citrus clementina* peels were washed firstly in tap water, then three times in deionized water before drying on sunlight and milling the samples. Then, dried and milled peels were dispersed in Milli-Q water (in ratio 0.05 g/mL) and ultrasonicated for 30 min. The obtained mixture was filtered, and 20 mL of the extract was transferred to a 50 mL of Teflon-lined stainless steel autoclave (Parr Instrument Company, Moline, IL, USA) and heated up to 180 °C for 12 h in an air oven for obtaining undoped and reference CQDs designated as pure CQDs. The preparation of N-doped CQDs included dissolving amino acids glycine (Gly, MW = 75.07 g/mol) or arginine (Arg, MW = 174.20 g/mol) in 5 mL of Milli-Q water (10 mg/mL), and overall 15 mL of a citrus extract with 5 mL of amino acid was transferred into Teflon-lined autoclave and heated at 180 °C for 12 h in an air oven. Finally, prepared CQDs were centrifuged at 15000 rpm, filtered via Nylon syringe filter (pore 0.2 μm; Agilent Technologies, Palo Alto, CA, USA), and dialyzed against Milli-Q water for 48 h which was found as a sufficient dialysis time for obtaining purified fluorescent samples. The purified solutions of CQDs were stored in the dark at 4°C and concentrated by vacuum rotary evaporator or dried by lyophilization procedure for further analysis. The schematic illustration of the CQDs synthesis discussed in this study is shown in Figure 8.

### 3.4. HPLC Analysis of the Dialyzed CQDs

In order to analyse the purification degree of prepared pure CQDs, the Agilent 1260 Infinity II HPLC system (Analytical Instruments, CA, USA) with separation on a ZORBAX Eclipse Plus C18 (Agilent Technologies, Palo Alto, CA, USA) column (100 × 4.6 mm, 5 m) was employed with methanol as phase A, and 10 mM of acetic acid and 25 mL of aqueous solution of NaCl with pH = 2.5 (adjusted using aqueous HCl solution) as phase B, according to the method developed by Chen et al. [28]. The gradient eluation protocol was as follows: from 0–25 min—10% of phase A + 90% of phase B; 25–45 min—a linear increase in phase A from 10% to 90%; and 45–70 min—90% of phase A + 10% of phase B. A sample volume of 150 μL was used for analysis, and the flow rate was maintained at 1.0 mL/min. The purification efficiency was investigated in three stages: (a) before dialysis; (b) after 24 h; and (c) after 48 h.

### 3.5. Quantum Yield (QY) Measurements

Fluorescence quantum yield using quinine sulphate as the reference standard (dissolved in 0.1 M H_2_SO_4_ solution; 54% at 360 nm) was measured and calculated according to the following Equation (1):

(1) $$\varphi_{CQD}=\varphi_{QS}\left[\frac{I_{CQD}}{A_{CQD}}\right]\left[\frac{A_{QS}}{I_{QS}}\right]\left[\frac{\eta_{CQD}^{2}}{\eta_{QS}^{2}}\right]$$

where *φ* designates quantum yield, *I* integrated fluorescence intensity under emission spectrum, *A* designates absorbance at the excitation wavelength, and η denotes refractive index (1.33 for aqueous solutions). The subscripts “CQD“ and “QS“ refer to the prepared samples of carbon quantum dots and quinine sulphate standard, respectively. It is noteworthy to mention that optical absorbency values were maintained at <0.1 to minimize the reabsorption effect. 

### 3.6. Cell Culturing and Antiproliferative Activity Assessment

The carcinoma cell lines HepG2 (hepatocellular carcinoma), MCF-7 (breast adenocarcinoma, metastatic), HCT-116 (colorectal carcinoma), CFPAC-1 (cystic fibrosis pancreatic adenocarcinoma, metastatic), and HFF-1 (human foreskin fibroblasts) (ATCC, American Type Culture Collection) were cultured in a humidified atmosphere with 5% CO_2_ at 37˚C as monolayers. The cells were maintained in Dulbecco’s modified Eagle medium (DMEM), supplemented with 10% fetal bovine serum (FBS), 2 mM of L-glutamine, 100 U/mL of penicillin, and 100 μg/mL of streptomycin. The cells were seeded into standard 96-well microtiter plates on day 0, at a seeding density of 3000 or 5000 cells per well according to the doubling time of cell lines. Freshly prepared five serial dilutions (0.01 to 100 µg/mL) of tested extracts were then added, and cells were further incubated for 72 h. After 72 h of incubation, the cell growth rate was evaluated by performing the MTT assay, according to the manufacturer’s instructions. The absorbance was measured at 570 nm (TECAN SUNRISE Microtiter plate reader, Männedorf, Switzerland) and transformed into a cell percentage growth (PG) using the formulas proposed by the National Institutes of Health (NIH) [57]. This method relies on comparing the growth of treated cells with untreated cells in control wells on the same plate, therefore presenting the results as a percentile difference from the calculated expected values. The IC_50_ and LC_50_ values (if applicable) for each compound were calculated from dose-response curves using linear regression analysis by fitting the mean test concentrations that give percentage growth values above and below the reference value. Each test point was performed in quadruplicate in two individual biological experiments.

### 3.7. Antioxidant Activity of CQDs and N-CQDs

A DPPH (2,2-diphenyl-1-picrylhydrazyl) assay was used to determine the antioxidant activity of the prepared CQDs with several modifications of the protocol reported by Sachdev and Gopinath [43]. Precisely, a series of diluted CQDs solutions were prepared in a concentration range from 0 to 100 µg/mL (0.2 mL) and mixed with 50 µM of methanolic solution of a DPPH scavenging radical (1.0 mL). The samples were kept in the dark for 30 min before measurements. The absorbance values were measured in triplicate at 517 nm using UV-Vis spectrophotometer, and antioxidant activity, compared to control blank samples, was calculated according to the following Equation (2):

(2) $$\%\;DPPH\;activity=\frac{\left(A_{DPPH}+A_{B}\right)-A_{S}}{A_{DPPH}}\times 100\%$$

where *A_B_* and *A_S_* are control samples without the addition of CQDs, and *A_DPPH_* are samples with added CQDs, respectively.

For the antioxidant activity assessment, aqueous extract of *Citrus clementina* peel was prepared by an ultrasound-assisted extraction technique following the procedure whereby 1 g of dried citrus peel suspended in 20 mL of Milli-Q water was sonicated for 1 h at 70 °C. The obtained extract was filtered through a filter paper and stored at 4 °C. The extraction was performed in an ultrasound-bath Elma, Elmasonic P 70 H, with a frequency of 37 kHz and a power of 50 W.

### 3.8. Measurements for Selective and Sensitive Fe^3+^ Detection

In order to evaluate the performance of synthesized CQDs toward ion sensing, ascorbic acid (AA) and overall nine different metal ions were studied for selectivity: Ca^2+^, Cu^2+^, Fe^3+^, K^+^, Hg^2+^, Mg^2+^, Al^3+^, Mn^2+^, Na^+^ ions. All metal ion stock solutions (100 µmol dm^−3^) were prepared by respective metals salts. The selectivity and sensibility studies were carried out with as-prepared CQD@Arg at a concentration of 25 µg/mL. The sensibility study for Fe^3+^ detection was performed at different Fe^3+^ concentrations ranging from 0.5–300 µmol dm^−3^ using fluorescence spectroscopy at the optimum excitation wavelength of λ_EX_ = 360 nm. Briefly, 100 µL of CQD@Arg solution (γ = 25 µg/mL) was added to 900 µL of Fe^3+^ salt solution of different concentrations for an overall probe volume of 1 mL. The selectivity investigation using the above-listed metal ions and AA was performed in a similar manner, as described above.

## 4. Conclusions

This work reports biomass-derived CQDs modified with amino acids Gly and Arg, resulting in a successful nitrogen doping of CQDs. The prepared samples containing nanosized particles (of the average size = 8.52 ± 1.72 nm) possessed good biocompatibility, satisfactory optical and chemical properties, and interesting biological activities. The prepared CQD@Gly exhibited the specific antiproliferative effect against pancreatic cancer cell line CFPAC-1, and CQD@Arg showed strong antioxidant activity (81.39 ± 0.39%) at a low concentration of 100 µg/mL. The highest quantum yield was determined with CQD@Arg, which was further investigated for the Fe^3+^ ion sensing and bioimaging. The developed model was described by an exponential function with a suitable coefficient of determination of *R*^2^ = 0.9891, while the linear range was determined in the concentration range from 7 µmol dm^−3^ to 50 µmol dm^−3^ with a determined limit of detection of LOD = 4.57 ± 0.27 µmol dm^−3^ and limit of quantification of LOQ = 15.24 ± 0.89 µmol dm^−3^. These findings could demonstrate the potential application of the prepared CQDs in bioimaging and ion sensing as a fluorescent probe with antioxidative or specific antitumor effects. The presented study may represent a novel and useful approach for efficient utilization of the waste for practical applications, including those in biomedicine and analytical chemistry.

## Figures and Tables

**Table 1:** Antiproliferative activity in vitro for samples of pure CQD, CQD@Arg, and CQD@Gly presented as IC_50_ values (μg/mL) ± standard deviation. Results from experiments carried out on human fibroblasts HFF-1 are presented for each biological replicate separately, while results for tumour cell lines are presented as means from three consecutive experiments.

Sample	Cell Line IC_50_ (µg/mL) ^1^					
	HepG2	CFPAC-1	MCF-7	HCT-116	HFF-1	
Pure CQD	>100	>100	>100	>100	1st experiment	20.59 ± 0.02
					2nd experiment	1.50 ± 0.02
					3rd experiment	>100
CQD@Arg	>100	>100	>100	>100	1st experiment	7.85 ± 0.02
					2nd experiment	*Proliferative effect*
					3rd experiment	>100
CQD@Gly	>100	6.91 ± 0.81	>100	>100	1st experiment	0.46 ± 0.01
					2nd experiment	*Proliferative effect*
					3rd experiment	>100

^1^ IC_50_; 50% inhibitory concentration, or compound concentration required to inhibit tumour cell proliferation by 50%.

**Table 2:** Comparison of the biomass-derived CQDs used for the detection of Fe^3+^ ions with some reported works.

No.	Carbon Precursor	Linear Range (μM)	LOD (μM)	Reported Applications	Reference
1	Coriander leaves	0−6	0.40	Cellular imaging, antioxidant activity	[43]
2	Hydrogenated rosin	0−60	6.16	Cellular imaging	[44]
3	Sweet potato	1−100	0.32	Cellular imaging	[45]
4	Blueberry	12.5−100	9.97	-	[46]
5	Hamburger sandwich leftover	12.5−100	32.0	-	[47]
6	Cranberry beans	30-600	9.55	-	[48]
7	Kiwi fruit peel	5-25	0.85	-	[49]
8	Sugarcane molasses	0−100	1.46	Cellular imaging, sensing of sunset yellow	[50]
9	*Chionanthus retusus* fruit	0−2	70.0	Bioimaging	[51]
10	Dwarf banana peel	5-25	0.66	Bioimaging, fluorescent ink	[52]
11	Apple juice	1-25	0.90	Bioimaging	[53]
12	*Citrus clementina* peel	7−50	4.57	Cellular imaging, antitumor and antioxidant activity, fluorescent ink	This work

## Data Availability

Data is contained within the article and Supplementary Materials.

## Supplementary Materials

The following are available online at https://www.mdpi.com/article/10.3390/ph14090857/s1, Figure S1: The UV-HPLC chromatograms of pure CQD retentate (a) before dialysis, (b) after 24 h, and (c) after 48 h, Figure S2: EDS mapping of nitrogen for (a) CQD@Gly and (b) CQD@Arg, Figure S3: XRD patterns of (a) pure CQD and (b) CQD@Gly, Figure S4: Integrated fluorescence intensity vs. absorbance of CQDs samples (recorded at λ_EX_=360 nm); calculated quantum yield (QY) of synthesized samples of CQDs using quinine sulfate as reference standard (*φ*_QS_ = 54%), Figure S5: Optical characterization of pure CQD: (a) Fluorescence emission spectra of pure CQD at different excitation wavelengths ranging from 320 nm to 440 nm (increments of 20 nm); (b) normalized fluorescence emission spectra, (c) stability of pure CQD in the high ionic medium of NaCl (0-1.0 mol dm^−3^; λ_EX_ = 360 nm. λ_EM_ = 445 nm); (d) effect of pH on fluorescence intensity (λ_EX_ = 360 nm, λ_EM_ = 445 nm), Figure S6: Optical characterization of CQD@Gly: (a) Fluorescence emission spectra of CQD@Gly at different excitation wavelengths ranging from 320 nm to 440 nm (increments of 20 nm); (b) normalized fluorescence emission spectra, (c) stability of CQD@Gly in the high ionic medium of NaCl (0–1.0 mol dm^−3^; λ_EX_ = 360 nm, λ_EM_ = 430 nm); (d) effect of pH on fluorescence intensity (λ_EX_ = 360 nm, λ_EM_ = 430 nm), Table S1: Photographs of prepared CQDs with calculated quantum yields under (a) daylight and (b) under UV light (365 nm). The photograph (c) shows text written using CQD@Arg under UV light (365 nm), Table S2: Elemental analysis of as-prepared CQDs.

## References

[1] Šafranko S., Goman D., Stanković A., Medvidović-Kosanović M., Moslavac T., Jerković I., Jokić S. (2021). An Overview of the Recent Developments in Carbon Quantum Dots—Promising Nanomaterials for Metal Ion Detection and (Bio)Molecule Sensing. Chemosensors.

[2] Ma X., Zhong W., Zhao J., Suib S., Lei Y. (2020). “Self-heating” enabled one-pot synthesis of fluorescent carbon dots. Eng. Sci..

[3] Carbonaro C.M., Corpino R., Salis M., Mocci F., Thakkar S.V., Olla C., Ricci P.C. (2019). On the Emission Properties of Carbon Dots: Reviewing Data and Discussing Models. C.

[4] Wang X., Feng Y., Dong P., Huang J. (2019). A Mini Review on Carbon Quantum A Mini Review on Carbon Quantum Dots: Preparation, Properties, and Electrocatalytic Application. Front. Chem..

[5] Kumar K., Yadav A.N., Kumar V., Vyas P., Dhaliwal H.S. (2017). Food waste: A potential bioresource for extraction of nutraceuticals and bioactive compounds. Bioresour. Bioprocess..

[6] Mahato N., Sharma K., Sinha M., Baral E., Koteswararao R., Dhyani A., Hwan Cho M., Cho S. (2020). Bio-sorbents, industrially important chemicals and novel materials from citrus processing waste as a sustainable and renewable bioresource: A review. J. Adv. Res..

[7] Jokić S., Molnar M., Cikoš A.-M., Jakovljević M., Šafranko S., Jerković I. (2019). Separation of selected bioactive compounds from orange peel using the sequence of supercritical CO_2_ extraction and ultrasound solvent extraction: Optimization of limonene and hesperidin content. Sep. Sci. Technol..

[8] Tadesse A., Hagos M., RamaDevi D., Basavaiah K., Belachew N. (2020). Fluorescent-Nitrogen-Doped Carbon Quantum Dots Derived from Citrus Lemon Juice: Green Synthesis, Mercury(II) Ion Sensing, and Live Cell Imaging. ACS Omega.

[9] Gao R., Wu Z., Wang L., Liu J., Deng Y., Xiao Z., Fang J., Liang Y. (2020). Green Preparation of Fluorescent Nitrogen-Doped Carbon Quantum Dots for Sensitive Detection of Oxytetracycline in Environmental Samples. Nanomaterials.

[10] Xiao-Yan W., Xue-Yan H., Tian-Qi W., Xu-Cheng F. (2019). Crown daisy leaf waste–derived carbon dots: A simple and green fluorescent probe for copper ion. Surf. Interface Anal..

[11] Sun X., Liu Y., Niu N., Chen L. (2019). Synthesis of molecularly imprinted fluorescent probe based on biomass-derived carbon quantum dots for detection of mesotrione. Anal. Bioanal. Chem..

[12] Atchudan R., Jebakumar Immanuel Edison T.N., Shanmugam M., Perumal S., Somanathan T., Lee Y.R. (2020). Sustainable synthesis of carbon quantum dots from banana peel waste using hydrothermal process for in vivo bioimaging. Phys. E Low Dimens. Syst. Nanostruct..

[13] Su R., Wang D., Liu M., Yan J., Wang J.X., Zhan Q., Pu Y., Foster N.R., Chen J.F. (2018). Subgram-Scale Synthesis of Biomass Waste-Derived Fluorescent Carbon Dots in Subcritical Water for Bioimaging, Sensing, and Solid-State Patterning. ACS Omega.

[14] Pramanik S., Chatterjee S., Kumar G.S., Devi P.S. (2018). Egg-shell derived carbon dots for base pair selective DNA binding and recognition. Phys. Chem. Chem. Phys..

[15] Chauhan P., Saini J., Chaudhary S., Bhasin K.K. (2021). Sustainable synthesis of carbon dots from agarose waste and prospective application in sensing of L-aspartic acid. Mater. Res. Bull..

[16] Liang Z., Zeng L., Cao X., Wang Q., Wang X., Sun R. (2014). Sustainable carbon quantum dots from forestry and agricultural biomass with amplified photoluminescence by simple NH_4_OH passivation. J. Mater. Chem. C.

[17] Dimos K. (2016). Carbon Quantum Dots: Surface Passivation and Functionalization. Curr. Org. Chem..

[18] Kou X., Jiang S., Park S.-J., Meng L. (2020). A Review: Recent Advances in Preparations and Applications of Heteroatom-Doped Carbon Quantum Dots. Dalton Trans..

[19] Qi H., Teng M., Liu M., Liu S., Li J., Yu H., Teng C., Huang Z., Liu H., Shao Q. (2019). Biomass-derived nitrogen-doped carbon quantum dots: Highly selective fluorescent probe for detecting Fe^3+^ ions and tetracyclines. J. Colloid Interface Sci..

[20] Goswami T., Rolfs A., Hediger M.A. (2002). Iron transport: Emerging roles in health and disease. Biochem. Cell Biol..

[21] Chung J.Y., Kim H.S., Song J. (2018). Iron metabolism in diabetes-induced Alzheimer’s disease: A focus on insulin resistance in the brain. BioMetals.

[22] Kim Y.O., Chung H.J., Kong H.S., Choi D.-W., Cho D.-H. (1999). The application of ion chromatographic method for bioavailability and stability test of iron preparations. Arch. Pharm. Res..

[23] Rivas-Cantu R.C., Jones K.D., Mills P.L. (2013). A citrus waste-based biorefinery as a source of renewable energy: Technical advances and analysis of engineering challenges. Waste Manag. Res..

[24] Su H., Bi Z., Ni Y., Yan L. (2019). One-pot degradation of cellulose into carbon dots and organic acids in its homogeneous aqueous solution. Green Energy Environ..

[25] Sakdaronnarong C., Sangjan A., Boonsith S., Kim D.C., Shin H.S. (2020). Recent Developments in Synthesis and Photocatalytic Applications of Carbon Dots. Catalysts.

[26] Feng Z., Li Z., Zhang X., Shi Y., Zhou N. (2017). Nitrogen-Doped Carbon Quantum Dots as Fluorescent Probes for Sensitive and Selective Detection of Nitrite. Molecules.

[27] Wu P., Li W., Wu Q., Liu Y., Liu S. (2017). Hydrothermal synthesis of nitrogen-doped carbon quantum dots from microcrystalline cellulose for the detection of Fe^3+^ ions in an acidic environment. RSC Adv..

[28] Chen C.-Y., Tsai Y.-H., Chang C.-W. (2019). Evaluating the dialysis time required for carbon dots by HPLC and the properties of the carbon dots after HPLC fractionation. New J. Chem..

[29] Li Y., Zhong X., Rider A., Furman S., Ostrikov K. (2014). Fast, energy-efficient synthesis of luminescent carbon quantum dots. Green Chem..

[30] Cai A., Wang Q., Chang Y., Wang X. (2017). Graphitic carbon nitride decorated with S,N co-doped graphene quantum dots for enhanced visible-light-driven photocatalysis. J. Alloy Compd..

[31] Qiang R., Yang S., Hou K., Wang J. (2019). Synthesis of carbon quantum dots with green luminescence from potato starch. New J. Chem..

[32] Jing S., Zhao Y., Sun R., Zhong L., Peng X. (2019). Facile and High-Yield Synthesis of Carbon Quantum Dots from Biomass-Derived Carbons at Mild Condition. ACS Sustain. Chem. Eng..

[33] Edison T.N.J.I., Atchudan R., Sethuraman M.G., Shim J.-J., Lee Y.R. (2016). Microwave assisted green synthesis of fluorescent N-doped carbon dots: Cytotoxicity and bio-imaging applications. J. Photochem. Photobiol. B Biol..

[34] Zhang Q., Liang J., Zhao L., Wang Y., Zheng Y., Wu Y., Jiang L. (2020). Synthesis of Novel Fluorescent Carbon Quantum Dots from *Rosa roxburghii* for Rapid and Highly Selective Detection of o-nitrophenol and Cellular Imaging. Front. Chem..

[35] Wang J., Wang J., Xiao W., Geng Z., Tan D., Wei L., Li J., Xue L., Wang X., Zhu J. (2020). Lignin-derived red-emitting carbon dots for colorimetric and sensitive fluorometric detection of water in organic solvents. Anal. Methods.

[36] Day R.M., Suzuki Y.J. (2005). Cell Proliferation, Reactive Oxygen and Cellular Glutathione. Dose-Response.

[37] Ji Z., Sheardy A., Zeng Z., Zhang W., Chevva H., Allado K., Yin Z., Wei J. (2019). Tuning the Functional Groups on Carbon Nanodots and Antioxidant Studies. Molecules.

[38] Zhu J., Chu H., Wang T., Wang C., Wei Y. (2020). Fluorescent probe based nitrogen doped carbon quantum dots with solid-state fluorescence for the detection of Hg^2+^ and Fe^3+^ in aqueous solution. Microchem. J..

[39] Issa M.A., Abidin Z.Z., Sobri S., Rashid S.A., Mahdi M.A., Ibrahim N.A. (2020). Fluorescent recognition of Fe^3+^ in acidic environment by enhanced-quantum yield N-doped carbon dots: Optimization of variables using central composite design. Sci. Rep..

[40] Lv P., Yao Y., Zhou H., Zhang J., Pang Z., Ao K., Cai Y., Wei Q. (2017). Synthesis of novel nitrogen-doped carbon dots for highly selective detection of iron ion. Nanotechnology.

[41] Anilbhai G.D., Desai M.L., Malek N.I., Kailasa S.K. (2020). Fluorescence detection of Fe^3+^ ion using ultra-small fluorescent carbon dots derived from pineapple (*Ananas comosus*): Development of miniaturized analytical method. J. Mol. Struct..

[42] Jiang X., Qin D., Mo G., Feng J., Yu C., Mo W., Deng B. (2019). Ginkgo leaf-based synthesization of nitrogen-doped carbon quantum dots for highly sensitive detection of salazosulfapyridine in mouse plasma. J. Pharm. Biomed. Anal..

[43] Sachdev A., Gopinath P. (2015). Green synthesis of multifunctional carbon dots from coriander leaves and their potential application as antioxidants, sensors and bioimaging agents. Analyst.

[44] Zhou J., Ge M., Han Y., Ni J., Huang X., Han S., Peng Z., Li Y., Li S. (2020). Preparation of Biomass-Based Carbon Dots with Aggregation Luminescence Enhancement from Hydrogenated Rosin for Biological Imaging and Detection of Fe^3+^. ACS Omega.

[45] Shen J., Shang S., Chen X., Wang D., Cai Y. (2017). Facile synthesis of fluorescence carbon dots from sweet potato for Fe^3+^ sensing and cell imaging. Mater. Sci. Eng. C.

[46] Aslandaş A., Balcı N., Arık M., Şakiroğlu H., Onganer Y., Meral K. (2015). Liquid nitrogen-assisted synthesis of fluorescent carbon dots from Blueberry and their performance in Fe^3+^ detection. Appl. Surf. Sci..

[47] Ahn J., Song Y., Kwon J., Woo J., Kim H. (2019). Characterization of food waste-driven carbon dot focusing on chemical structural, electron relaxation behavior and Fe^3+^ selective sensing. Data Brief.

[48] Zulfajri M., Gedda G., Chang C., Chang Y., Huang G. (2019). Cranberry Beans Derived Carbon Dots as a Potential Fluorescence Sensor for Selective Detection of Fe^3+^ Ions in Aqueous Solution. ACS Omega.

[49] Atchudan R., Edison T.N.J.I., Perumal S., Vinodh R., Sundramoorthy A.K., Babu R.S., Lee Y.R. (2021). Leftover Kiwi Fruit Peel-Derived Carbon Dots as a Highly Selective Fluorescent Sensor for Detection of Ferric Ion. Chemosensors.

[50] Huang G., Chen X., Wang C., Zheng H., Huang Z., Chen D., Xie H. (2017). Photoluminescent carbon dots derived from sugarcane molasses: Synthesis, properties, and applications. RSC Adv..

[51] Atchudan R., Edison T.N.J.I., Chakradhar D., Perumal S., Shim J.-J., Lee Y.R. (2017). Facile green synthesis of nitrogen-doped carbon dots using *Chionanthus retusus* fruit extract and investigation of their suitability for metal ion sensing and biological applications. Sens. Actuators B Chem..

[52] Atchudan R., Edison T.N.J.I., Perumal S., Muthuchamy N., Lee Y.R. (2020). Hydrophilic nitrogen-doped carbon dots from biowaste using dwarf banana peel for environmental and biological applications. Fuel.

[53] Mehta V.N., Jha S., Basu H., Singhal R.K., Kailasa S.K. (2015). One-step hydrothermal approach to fabricate carbon dots from apple juice for imaging of mycobacterium and fungal cells. Sens. Actuators B Chem..

[54] Khan S., Gupta A., Verma N.C., Nandi C.K. (2015). Time-Resolved Emission Reveals Ensemble of Emissive States as the Origin of Multicolor Fluorescence in Carbon Dots. Nano Lett..

[55] Essner B.J., Kist J.A., Polo-Parada L., Baker G.A. (2018). Artifacts and Errors Associated with the Ubiquitous Presence of Fluorescent Impurities in Carbon Nanodots. Chem. Mater..

[56] Demchenko A.P. (2019). Excitons in Carbonic Nanostructures. C.

[57] Gazivoda T., Raić-Malić S., Krištafor V., Makuc D., Plavec J., Bratulić S., Kraljević-Pavelić S., Pavelić K., Naesens L., Andrei G. (2008). Synthesis, cytostatic and anti-HIV evaluations of the new unsaturated acyclic C-5 pyrimidine nucleoside analogues. Bioorg. Med. Chem..

