# Supplementary material for: Preparation of Multifunctional N-Doped Carbon Quantum Dots from Citrus clementina Peel: Investigating Targeted Pharmacological Activities and the Potential Application for Fe3+ Sensing
Source: Pharmaceuticals (Basel). 2021 Aug 27;14(9):857. doi: 10.3390/ph14090857 (PMC8465261; doi:10.3390/ph14090857)
Supplement: Supplementary file 1 [file pharmaceuticals-14-00857-s001.zip › pharmaceuticals-1351758-supplementary.pdf]

Supplementary Material for

Preparation of Multifunctional N-doped Carbon Quantum Dots from *Citrus clementina* Peel: Investigating Targeted Pharmacological Activities and the Potential Application for Fe^{3+} Sensing

Silvija Šafranko [1], Anamarija Stanković [2], Sugato Hajra [3], Hoe-Joon Kim [3], Ivica Strelec [1], Maja Dutour-Sikirić [4], Igor Weber [5], Maja Herak Bosnar [6], Petra Grbčić [7], Sandra Kraljević Pavelić [8], Aleksandar Széchenyi [9], Yogendra Kumar Mishra [10], Igor Jerković [11,*] and Stela Jokić [1,*]

[1] Faculty of Food Technology Osijek, University of Osijek, Franje Kuhača 18, 31000 Osijek, Croatia; silvija.safranko@ptfos.hr (S.Š.); ivica.strelec@ptfos.hr (I.S.)
[2] Department of Chemistry, University of Osijek, Ulica cara Hadrijana 8/A, 31000 Osijek, Croatia; astankovic@kemija.unios.hr
[3] Department of Robotics Engineering, Daegu Gyeongbuk Institute of Science & Technology (DGIST), Daegu 42988, Korea; sugatohajra@dgist.ac.kr (S.H.); joonkim@dgist.ac.kr (H.-J.K.)
[4] Division of Physical Chemistry, Ruđer Bošković Institute, 10000 Zagreb, Croatia; maja.dutour.sikiric@irb.hr
[5] Division of Molecular Biology, Ruđer Bošković Institute, 10000 Zagreb, Croatia; iweber@irb.hr
[6] Division of Molecular Medicine, Ruđer Bošković Institute, 10000 Zagreb, Croatia; mherak@irb.hr
[7] Department of Biotechnology, University of Rijeka, Radmile Matejčić 2, 51000 Rijeka, Croatia; petra.grbcic@biotech.uniri.hr
[8] Faculty of Health Studies, University of Rijeka, Viktora Cara Emina 5, 51000 Rijeka, Croatia; sandrakp@uniri.hr
[9] Institute of Pharmaceutical Technology and Biopharmacy, Faculty of Pharmacy, University of Pécs, H-7624 Pécs, Hungary; szealex@gamma.ttk.pte.hu
[10] Mads Clausen Institute, NanoSYD, University of Southern Denmark, Alsion 2, 6400 Sønderborg, Denmark; mishra@mci.sdu.dk
[11] Department of Organic Chemistry, Faculty of Chemistry and Technology, University of Split, Ruđera Boškovića 35, 21000 Split, Croatia
* Correspondence: igor@ktf-split.hr (I.J.); sjokic@ptfos.hr (S.J.); Tel.: +385-21-329-461 (I.J.); +385-31-224-333 (S.J.)

Content of listed material

Figure S1. UV-HPLC chromatograms of Pure CQD retentate before dialysis (a), after (b) 24 hours and (c) 48 hours.

Figure S2. EDS mapping of nitrogen for a) CQD@Gly and b) CQD@Arg

Figure S3. XRD patterns of a) Pure CQD and b) CQD@Gly

Figure S4. Integrated fluorescence intensity vs. absorbance of CQDs samples (recorded at λ_{EX}=360 nm); calculated quantum yield (QY) of synthesized samples of CQDs using quinine sulfate as reference standard (φ_{QS} = 54 %)

Figure S5. Optical characterization of Pure CQD: a) Fluorescence emission spectra of Pure CQD at different excitation wavelengths ranging from 320 nm to 440 nm (increments of 20 nm); b) normalized fluorescence emission spectra, c) stability of Pure CQD in the high ionic medium of NaCl (0-1.0 mol dm^{-3}; λ_{EX} = 360 nm. λ_{EM} = 445 nm); d) effect of pH on fluorescence intensity (λ_{EX} = 360 nm, λ_{EM} = 445 nm)

Figure S6. Optical characterization of CQD@Gly: a) Fluorescence emission spectra of CQD@Gly at different excitation wavelengths ranging from 320 nm to 440 nm (increments of 20 nm); b) normalized fluorescence emission spectra, c) stability of CQD@Gly in the high ionic medium of NaCl (0-1.0 mol dm^{-3}; λ_{EX} = 360 nm, λ_{EM} = 430 nm); d) effect of pH on fluorescence intensity (λ_{EX} = 360 nm, λ_{EM} = 430 nm)

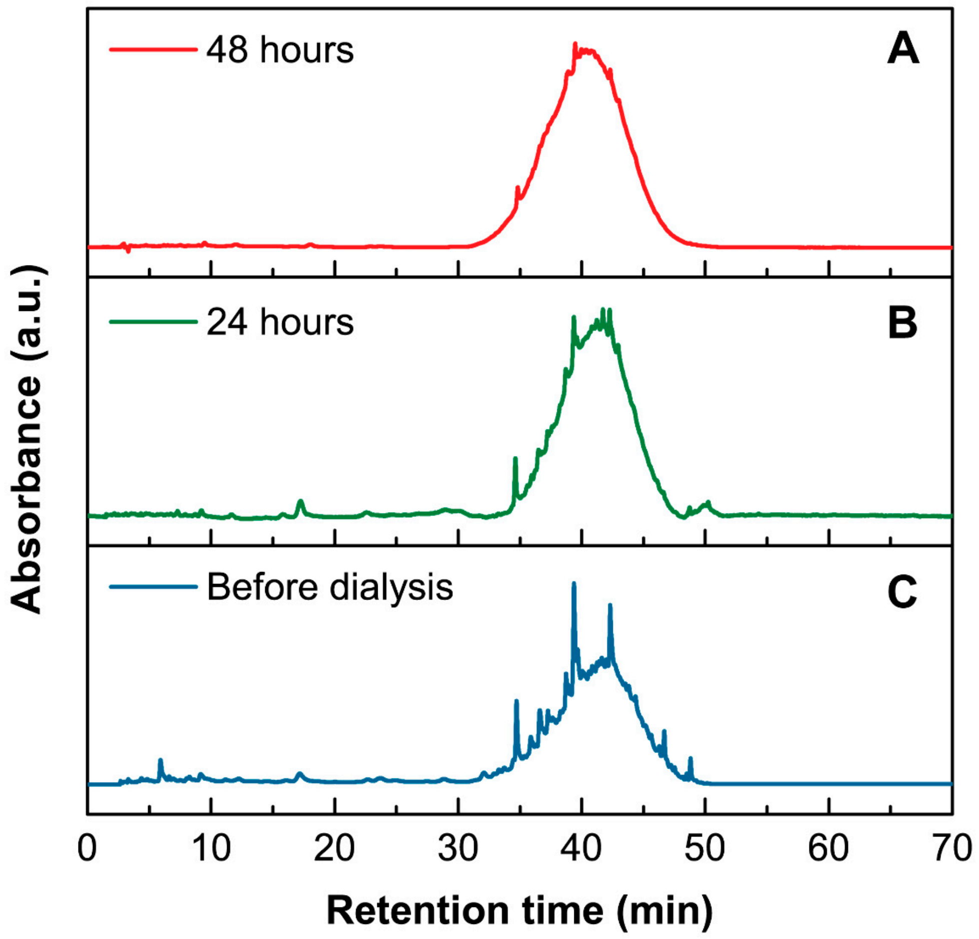
48 hours
A
24 hours
B
Before dialysis
C
Absorbance (a.u.)
0
10
20
30
40
50
60
70
Retention time (min)

Figure S1.

A) CQD@Gly **B) CQD@Arg**

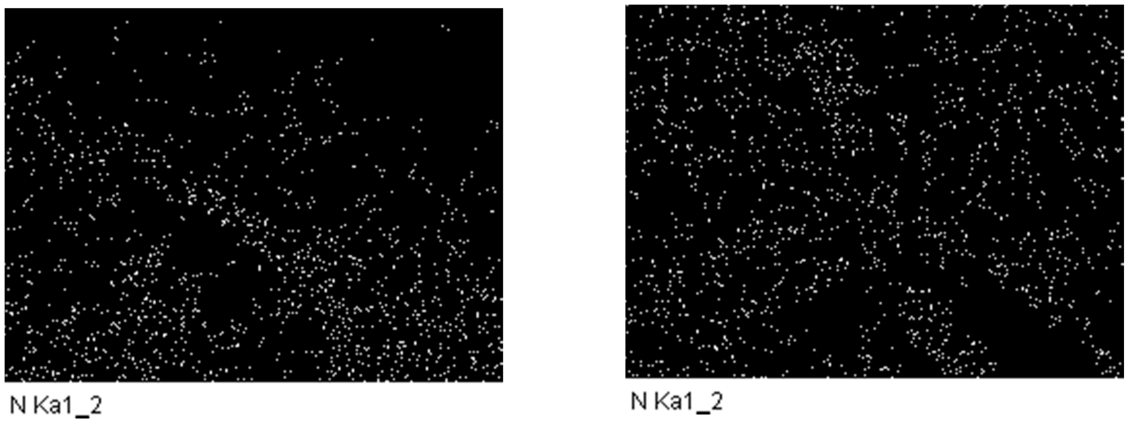

Figure S2.

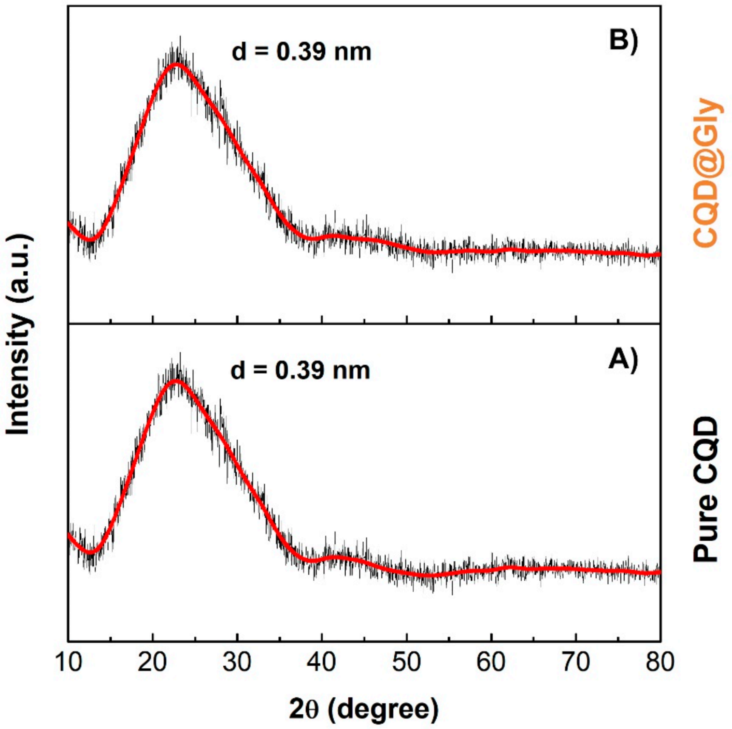

Figure S3.

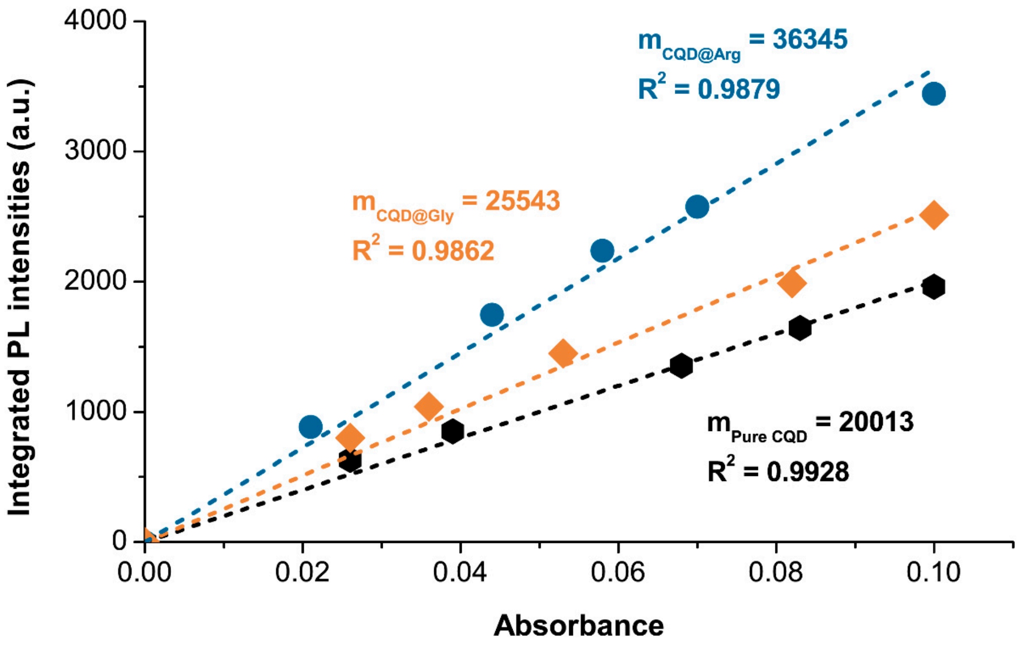

m CQD@Arg = 36345
R2 = 0.9879
m CQD@Gly = 25543
R2 = 0.9862
m Pure CQD = 20013
R2 = 0.9928
Integrated PL intensities (a.u.)
Absorbance
0
1000
2000
3000
4000
0.00
0.02
0.04
0.06
0.08
0.10

Figure S4.

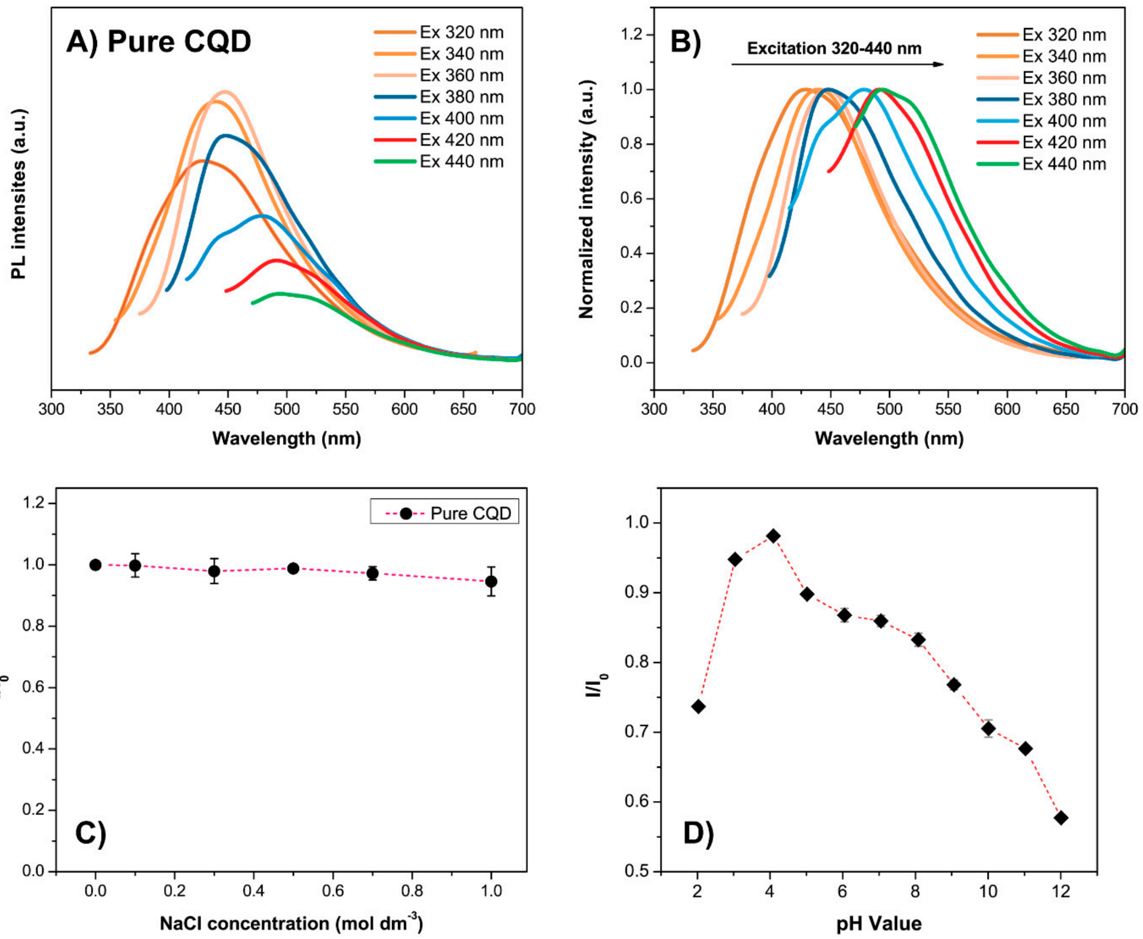
A) Pure CQD
Ex 320 nm
Ex 340 nm
Ex 360 nm
Ex 380 nm
Ex 400 nm
Ex 420 nm
Ex 440 nm
PL intensites (a.u.)
Wavelength (nm)
B)
Excitation 320-440 nm
Normalized intensity (a.u.)
C)
Pure CQD
I/I0
NaCl concentration (mol dm-3)
D)
pH Value

Figure S5.

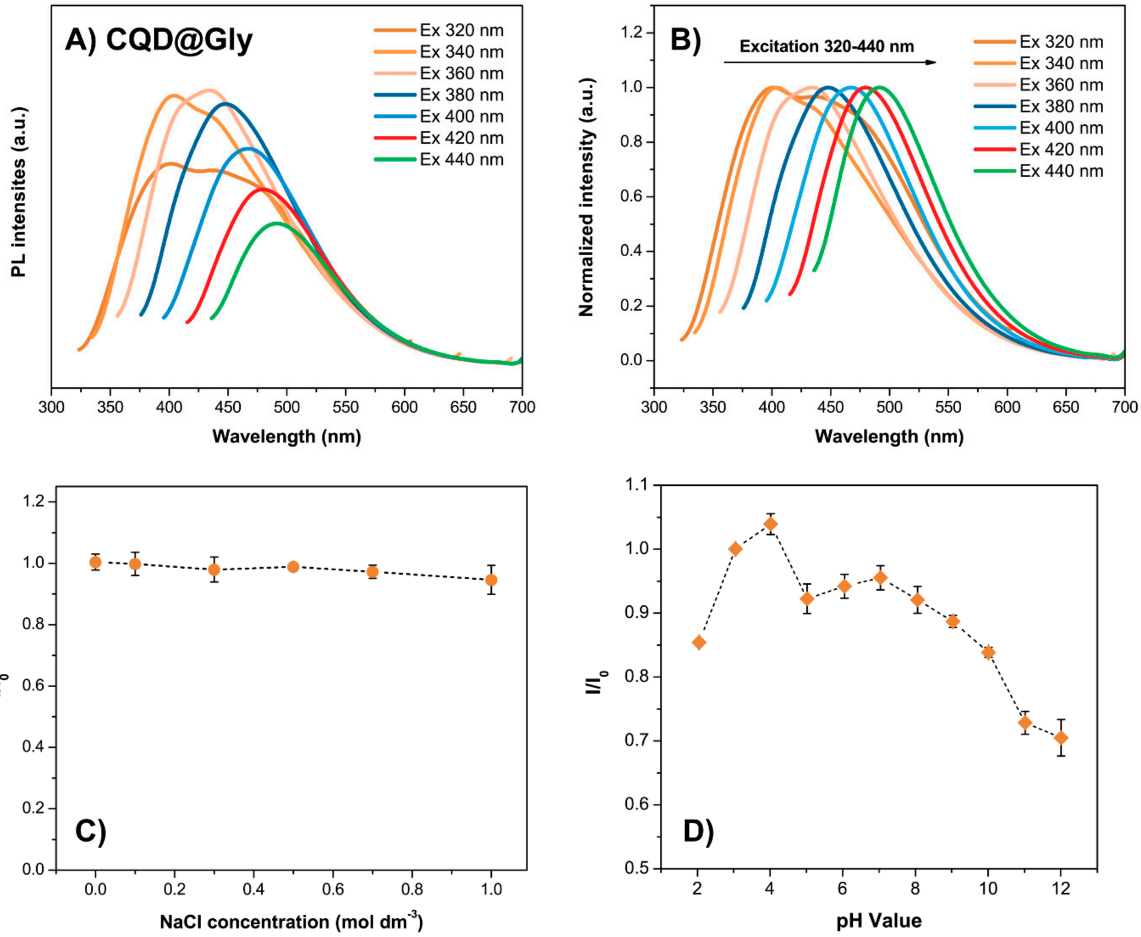
A) CQD@Gly
Ex 320 nm
Ex 340 nm
Ex 360 nm
Ex 380 nm
Ex 400 nm
Ex 420 nm
Ex 440 nm
PL intensites (a.u.)
Wavelength (nm)
B)
Excitation 320-440 nm
Normalized intensity (a.u.)
C)
I/I0
NaCl concentration (mol dm-3)
D)
pH Value

Figure S6.

Table content

Table S1. Photographs of prepared CQDs with calculated quantum yields under a) daylight and b) under UV light (365 nm). The photograph (c) shows text written using CQD@Arg under UV light (365 nm)

Sample	A	B
**Pure CQD** (QY: 1.17 %)	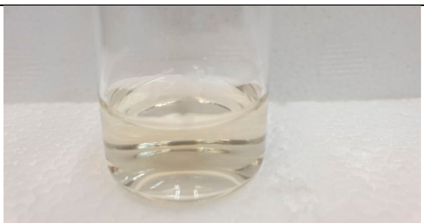	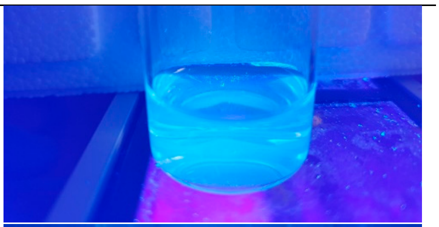
**CQD@Gly** (QY: 1.53 %)	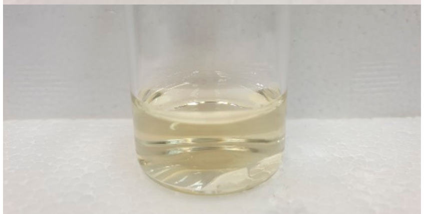	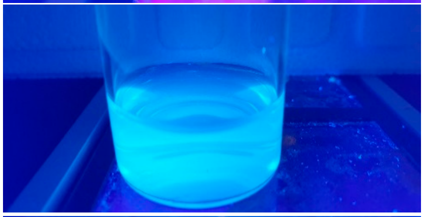
**CQD@Arg** (QY: 2.17 %)	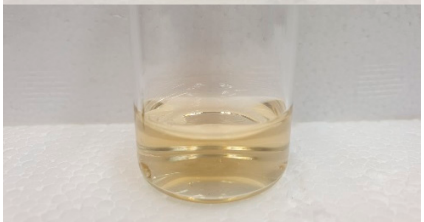	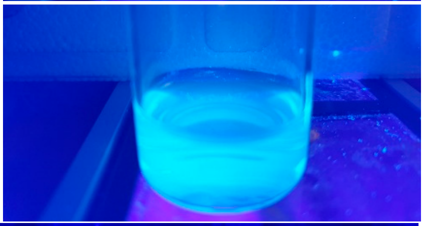
**Fluorescent Ink (C)**	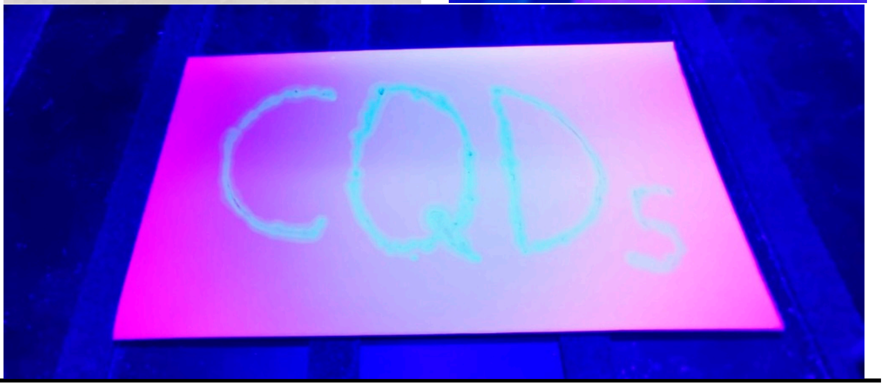	

Table S2. Elemental analysis of as-prepared CQDs

	Weight%			Atomic%		
Sample	C (%)	O (%)	N (%)	C (%)	O (%)	N (%)
**Pure CQD**	52.20	39.03	-	61.89	34.74	-
**CQD@Gly**	59.81	31.80	3.16	67.74	27.04	3.07
**CQD@Arg**	58.76	28.64	4.94	67.45	24.68	4.87
